# Poor Adherence to National and International Breastfeeding Duration Targets in an Australian Longitudinal Cohort

**DOI:** 10.1371/journal.pone.0054409

**Published:** 2013-01-30

**Authors:** Alexis J. Hure, Jennifer R. Powers, Catherine L. Chojenta, Julie E. Byles, Deborah Loxton

**Affiliations:** Research Centre for Gender Health and Ageing, School of Medicine and Public Health, University of Newcastle, Newcastle, Australia; The University of Hong Kong, Hong Kong

## Abstract

**Objectives:**

To report on the proportion and characteristics of Australian infants who are fed, and mothers who feed, in accordance with the national and international breastfeeding duration targets of six, 12 and 24 months. Furthermore, to examine the longitudinal breastfeeding duration patterns for women with more than one child.

**Methods:**

Breastfeeding duration data for 9773 children have been self-reported by a national sample of 5091 mothers aged 30–36 years in 2009, participating in the Australian Longitudinal Study on Women’s Health.

**Results:**

Only 60% of infants received the minimum recommended 6 months of breast milk, irrespective of breastfeeding exclusivity. Less than 30% of infants received any breast milk at 12 months, and less than 3% were breastfed to the international target of 24 months. Young, less educated, unmarried or low-income women were at an increased risk of premature breastfeeding cessation. For women with three or more children, nearly 75% of women who breastfed their first child for at least six months reached this breastfeeding duration target for their next two children.

**Conclusion:**

While national breastfeeding rates are typically evaluated in relation to the infant, a novel component of our study is that we have assessed maternal adherence to breastfeeding duration targets and the longitudinal feeding practices of women with more than one child. Separate evaluations of maternal and infant breastfeeding rates are important as they differ in their implications for public health policy and practice.

## Introduction

Of all preventive interventions, optimal breastfeeding of children under two years has the greatest potential to improve child survival [1]. More exclusive (up to six months) and longer duration breastfeeding shows the strongest associations with favourable health outcomes for both the mother and child [2]. For over a decade the World Health Organization has recommended that all infants be exclusively breastfed for the first six months, with continued breastfeeding to two years and beyond [3]. While Australian policies have been largely influenced by international standards [4], our current Infant Feeding Guidelines recommend breastfeeding continue to at least 12 months [5]. The Australian guidelines do, however, acknowledge that “substantial benefits may continue for two years and beyond” [5]. Breastfeeding duration is defined as the total length of time a child receives any breast milk, from initiation through to complete weaning.

National statistics on breastfeeding in Australia have recently been updated [6]. Like other developed countries [7], [8], Australia shows a high rate of breastfeeding initiation (96%) but poor continuation [6]. The rates of any breastfeeding were 75% at one month, 69% at four months, 60% at six months, 42% at seven to 12 months and 7% at 19–24 months [6]. However, the actual proportion of infants’ breastfed to the 12 and 24 month duration targets is not reported in the *2010 Australian National Infant Feeding Survey*. Earlier figures on national breastfeeding come from the Longitudinal Study of Australian Children, a birth cohort of more than 5000 infants from 2004. Their figures showed that only 30% of infants were breastfed to 12 months and 5% reached 24 months [2].

Limitations with study design, infrequent data collection, inconsistent breastfeeding definitions, and methodological discrepancies between surveys leave significant gaps in Australian breastfeeding literature. In particular, we are missing nationally representative data on the breastfeeding patterns of the same woman over her reproductive lifespan. Interestingly, the breastfeeding guidelines are written with a focus on how the infant is fed and this is typically reflected in evaluations of national breastfeeding rates. Another approach is to consider that interventions to enhance breastfeeding must be mediated via the mother, who may be responsible for feeding multiple children during her childbearing years. Hence women’s adherence to breastfeeding duration targets should also be assessed.

Breastfeeding duration data have been collected from a large sample of women aged 30–36 years, participating in the Australian Longitudinal Study on Women’s Health (ALSWH). The aims of the present study were to determine the:

1. proportions of children breastfed to the national and international duration targets of six, 12 and 24 months;
2. child-related characteristics associated with being breastfed to the duration targets;
3. proportions of mothers who breastfeed some or all of their children to the national and international duration targets of six, 12 and 24 months;
4. maternal characteristics associated with adhering to the breastfeeding duration targets; and
5. longitudinal breastfeeding duration patterns for women with more than one child.

## Methods

### Participants

Ethics approvals for the ALSWH were obtained from the Human Research Ethics Committees of the Universities of Newcastle and Queensland, and written informed consent was provided by participants. Women born between 1973 and 1978 (the 1973–78 cohort) were invited to participate in the baseline survey of the ALSWH in 1996 [9]. Potential participants were randomly selected, with intentional over-sampling of women living in rural and remote areas, from the Medicare database that includes all permanent residents in Australia [9]. At baseline, 14,247 women (41–42%) aged 18–23 years consented to participate [10]. Respondents were broadly representative of women of the same age in the 1996 Census, with some over-representation of more educated and Australian-born women [10]. Subsequent mailed surveys were sent to the 1973–78 cohort in 2000 (Survey 2), 2003 (Survey 3), 2006 (Survey 4) and 2009 (Survey 5).

Survey 5 responses were received from 58% (n = 8200) of those who completed the baseline survey in 1996. Compared to non-responders, more women who completed Survey 5 had never smoked (54% vs. 45%) and had 12 years or more education (70% vs. 65%) at baseline [11]. However, women who completed Survey 5 were not meaningfully different to non-responders in terms of age, marital status, or area of residence at baseline [11]. Despite losing women who smoked and were less educated, previous analyses have shown no serious bias in estimates of associations between socio-demographic and health characteristics caused by attrition [12]. Of the 8200 women who responded to Survey 5, 5241 provided data for 10,377 live births. Children were excluded from the analyses if they were multiple births (n = 300) or were missing breastfeeding data or date of birth (n = 304). Each mother had to have breastfeeding data for at least one live birth to be included. These analyses include data recalled at Survey 5 (completed in 2009 to 2010) for the remaining 9773 children, born to 5091 women between 1989 and 2010.

### Breastfeeding Duration

In 2009, women were asked ‘How many complete months have you breastfed each of your children?’ No measure of breastfeeding exclusivity was collected and data are limited to complete months of breastfeeding, meaning breastfeeding initiation could not be assessed. Women and children were categorised according to meeting the national (12 months) [5] and international (six and 24 months) [3] breastfeeding duration targets. Each mother’s adherence to breastfeeding duration targets was assessed for all of her singleton live-born children. Mothers were ‘fully adherent’ if they breastfed all their children according to each target, ‘partly adherent’ if they breastfed at least one but not all children according to each target, and ‘non-adherent’ if they did not breastfeed any of their children for six, 12, or 24 months or more. Children being breastfed and women who were currently breastfeeding at the time of the survey, who had not reached the breastfeeding duration target, were classed as ‘unknown’ when reporting the breastfeeding rates and were excluded from subsequent analyses. However, when breastfeeding had ceased before the six, 12, and 24 month targets, the children and mothers were included, even though the child may not have been six, 12 or 24 months of age.

### Other Measures

Child-related characteristics were assessed in relation to being breastfed according to the duration targets. These included the mother’s age at their birth (14–19, 20–24, 25–29, 30–36 years), birth order (1, 2, 3 or more), and preterm (not defined) and/or low birth weight (less than 2500 grams) or not. Maternal characteristics were also compared for women who breastfed their children according to the duration targets. The reproductive and socio-demographic characteristics included age at first birth (14–19, 20–24, 25–29, 30–36 years), number of births (1, 2, 3 or more), and level of education by 2009 (less than 12 years school; 12 years school; trade, certificate or diploma; university), relationship status (married; living in a *de facto* relationship; separated, divorced or widowed; single), whether women were very or extremely stressed about money (yes, no), and area of residence (living in or outside a major city).

### Statistical Analysis

The proportions of infants breastfed according to the duration targets of six, 12 or 24 months were calculated. The proportions of mothers who were fully, partly or non-adherent to each duration target for all their children were also calculated. Descriptive statistics were used to summarise the child-related and maternal characteristics associated with meeting the breastfeeding duration targets, and comparisons were made using χ^2^. The longitudinal breastfeeding patterns of mothers with two or more children were examined separately, again using descriptive statistics. Analyses were performed using SAS version 9.2 (SAS Institute, Cary, NC, USA). Based on the large sample size and multiple comparisons, p<0.01 was considered statistically significant.

## Results

Almost 34% of the women (n = 1723) had one child, 46% had two children (n = 2338) and 20% had between three and seven children (n = 1030). The mean age (standard deviation) at first birth was 27.7 (SD: 4.1) years. For mothers who had breastfed for at least one completed month (n = 4766) the average time since their last breastfeeding episode was 2.8 (SD: 3.2) years.

### Breastfeeding Duration for Each Child

**Figure 1:**
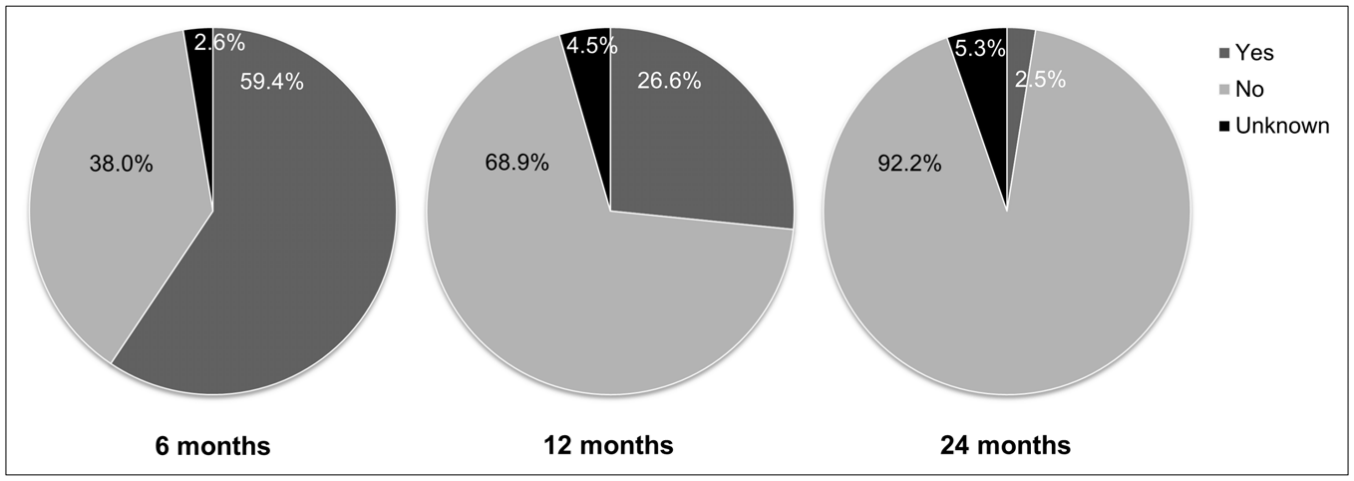
The proportion of Australian children (N = 9773) who were breastfed according to national and international duration targets of six, 12, and 24 months or more. ^a^Unknown: breastfeeding was ongoing at the time of the survey and the duration target had not been reached.

Children (N = 9773) were breastfed for between 0 and 63 months, with 9% (n = 920) not breastfed for one completed month. Figure 1 shows 59% of children (n = 5813) were breastfed to six months, 27% (n = 2596) were breastfed to 12 months, and only 3% (n = 241) were breastfed to two years or beyond.

Children who were born to younger mothers were less likely to be breastfed to duration target compared to children born to older women (Table 1). Being the first child or being born preterm and/or low birth weight also reduced the likelihood of receiving breast milk for six or 12 months. By the 24 month mark, with so few children being breastfed to this point, the only significant result remained for birth order.

**Table 1:** Characteristics of children by completed months of breastfeeding in line with the national and international duration targets.

	Completed months of breastfeeding								
	≥6 months			≥12 months			≥24 months		
	n = 5813 of 9523^a^	%	?^2^ p-value	n = 2596 of 9336 a	%	?^2^ p-value	n = 241 of 9252 a	%	?^2^ p-value
Mother’s age at birth (years)									
14–19	316	38.0	<.01	316	13.6	<.01	316	1.6	0.08
20–24	1450	51.5		1450	23.3		1450	3.2	
25–29	3844	62.5		3843	29.0		3839	2.9	
30–36	3913	65.0		3727	29.5		3647	2.2	
Birth order									
1	4919	59.4	<.01	4865	27.4	<.01	4828	2.2	<.01
2	3264	61.2		3187	26.7		3161	2.5	
≥3	1340	66.6		1284	32.1		1263	4.4	
Preterm and/or low birth weight									
Both	296	48.6	<.01	291	19.6	<.01	291	1.7	0.50
1	665	50.8		657	20.8		652	2.3	
0	8562	62.3		8388	28.6		8309	2.7	

a Children being breastfed at the time of the survey who had not reached the breastfeeding duration target were classed as ‘unknown’ and were excluded from these analyses. When breastfeeding had ceased children were included regardless age.

### Breastfeeding Duration by Each Mother

**Figure 2:**
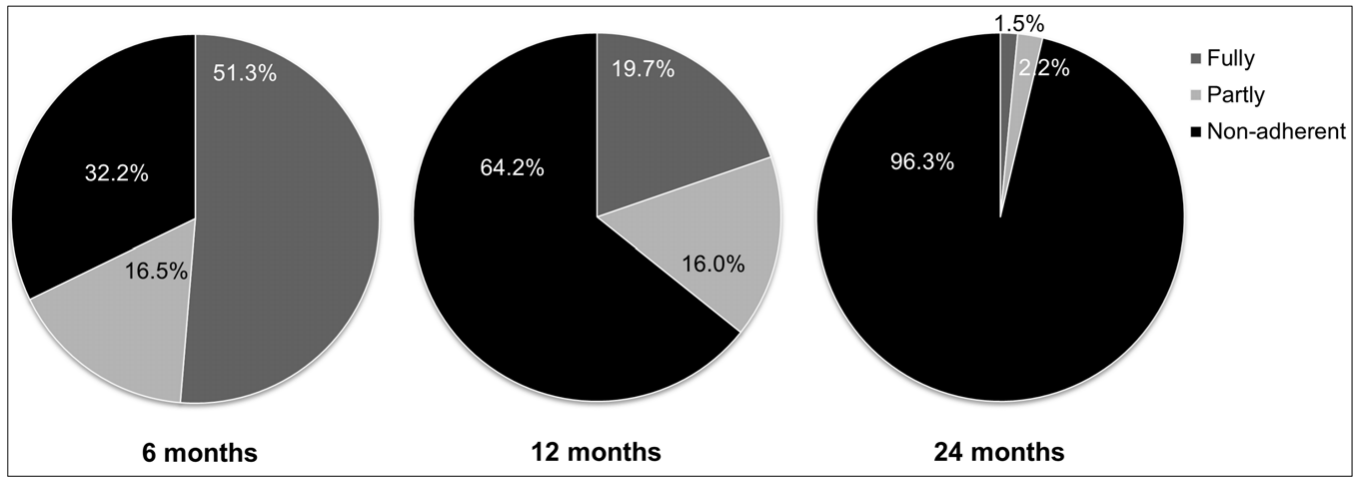
The proportion of Australian mothers (N = 4990) who were fully, partly or non-adherent to the breastfeeding duration targets of six, 12, and 24 months or more for all of their children.

Mothers’ (n = 4990) adherence to breastfeeding duration targets for all of their children is shown in Figure 2. Fifty-one percent were fully adherent to the breastfeeding guideline of six months or more for all of their children, 20% were fully adherent to the 12 month target, with only 2% fully adherent by 24 months. Nearly one third of mothers did not breastfeed any their children to six months or more and two thirds did not breastfeed for 12 months or more.

Proportionally, more than twice as many mothers aged 30–36 years at their first birth were fully adherent to the duration targets of six, 12 and 24 months or more, compared to women who had their first birth before the age of 20 (Table 2). Having had a greater number of children was associated with a lower proportion of mothers being fully adherent, but a higher proportion being partly adherent, at six, 12 and 24 months. Women who had a university degree were twice as likely to breastfeed all of their children (fully adherent) for at least six months compared to women who did not complete high school. Seventy percent of married women breastfed one or more children to at least six months (fully or partly adherent) compared to 51% of women who were single in 2009. On the other hand, five times as many single women met the 24 month duration target, compared to married women (proportionally), though the absolute numbers were small. Women who were not stressed about money were more likely to breastfed all of their children for six and 12 months compared with women who were stressed about money. Area of residence was not a significant predictor of breastfeeding duration at p<0.01.

**Table 2:** Characteristics of mothers by adherence to breastfeeding duration targets.

	≥6 months (n = 4990^a^)					≥12 months (n = 4936^a^)					≥24 months (n = 4899^a^)				
Characteristics in 2009		Fully adherent	Partly adherent	Non-adherent			Fully adherent	Partly adherent	Non-adherent			Fully adherent	Partly adherent	Non-adherent	
		n = 2558	n = 823	n = 1609	?^2^		n = 973	n = 792	n = 3171	?^2^		n = 72	n = 110	n = 4717	?^2^
	n	%	%	%	p-value	n	%	%	%	p-value	n	%	%	%	p-value
Mother’s age at first birth (years)															
14–19	294	26.2	33.3	40.5	<.01	294	8.2	22.8	69.1	<.01	294	0.7	4.8	94.6	<.01
20 – 24	941	40.3	26.9	32.8		941	14.1	23.0	62.9		941	1.9	4.0	94.1	
25 – 29	2147	53.1	17.2	29.7		2147	20.2	18.4	61.3		2145	1.3	2.2	96.5	
30 – 36	1608	59.8	6.3	33.8		1554	24.6	7.3	68.2		1519	1.6	0.7	97.7	
Number of children															
1	1719	57.4	0	42.6	<.01	1743	51.7	0	72.0	<.01	1732	2.6	0	97.4	<.01
2	2280	50.1	19.6	30.3		2236	16.5	19.9	63.6		2224	0.9	2.5	96.6	
≥3	991	43.3	38.0	18.7		957	12.1	36.2	51.7		943	0.7	5.8	93.4	
Education															
Less than 12 years school	466	33.2	23.0	43.8	<.01	466	10.9	20.4	68.7	<.01	465	0.7	3.9	95.5	<.01
12 years school	811	40.6	20.1	39.3		806	12.5	15.6	71.8		801	0.4	2.6	97.0	
Trade, certificate, diploma	1429	45.4	17.3	37.3		1420	16.0	16.0	68.0		1417	1.3	2.3	96.4	
University degree	2229	63.0	13.0	24.0		2189	26.9	15.2	57.9		2161	2.2	1.8	96.0	
Relationship status															
Single	174	43.7	7.5	48.8	<.01	174	19.5	9.2	71.3	<.01	171	6.4	1.8	91.8	<.01
Separated, divorced, widowed	306	38.9	20.9	40.2		306	14.0	18.3	67.7		306	1.6	3.3	95.1	
* De facto*	571	44.0	16.3	39.7		560	18.6	12.7	68.7		555	2.0	2.3	95.7	
Married	3921	53.6	16.5	29.9		3878	20.2	16.7	63.1		3849	1.2	2.2	96.6	
Very or extremely stressed about money															
Yes	1015	41.9	18.9	39.2	<.01	1012	16.1	13.7	70.2	<.01	1008	1.8	2.2	96.0	0.66
No	3962	53.7	15.9	30.4		3911	20.7	16.6	62.7		3878	1.4	2.2	96.4	
Area of residence															
Outside major cities	2532	49.8	17.8	32.4	0.02	2515	19.8	17.4	62.8	0.02	2503	1.5	2.5	96.0	0.51
Major cities	2458	52.8	15.1	32.1		2421	19.6	14.7	65.7		2396	1.4	2.0	96.6	

a Mothers breastfeeding at the time of the survey who had not reached the breastfeeding duration target were classed as ‘unknown’ and were excluded from these analyses. When breastfeeding had ceased mothers were included regardless of the child’s age.

Longitudinal breastfeeding patterns were examined for 3222 mothers with two or more children (Table 3) who had either reached the six month breastfeeding duration target or had ceased breastfeeding; n = 3145 at 12 months. Women were much more likely to breastfeed their second child for six months or more if they had breastfed their first child for at least six months. Eighty-four percent (n = 1634) of the 1951 women who had breastfed their first child, breastfed their second child for six months or more. In comparison, only 27% (n = 340) of the 1271 women who had not breastfed their first child for six months or more breastfed their second child for six months or more.

**Table 3:** Longitudinal patterns of breastfeeding duration^a^ for women with two or more children.

	First child	Second child (N = 3222)	n	%	Third child (N = 963)	n	%
***6 month target –*** ** completed** **months of breastfeeding**	0–5 months	0–5 months	931	73.2	0–5 months	189	49.6
					≥6 months	61	16.0
		≥6 months	340	26.8	0–5 months	23	6.0
					≥6 months	108	28.3
		N =	1271		N =	381	
	≥6 months	0–5 months	317	16.2	0–5 months	41	7.0
					≥6 months	42	7.2
		≥6 months	1634	83.8	0–5 months	68	11.7
					≥6 months	431	74.1
		N =	1951		N =	582	
	**First child**	**Second child (N = 3145)**	**n**	**%**	**Third child (N = 932)**	**n**	**%**
***12 month target –*** ** completed** **months of breastfeeding**	0–11 months	0–11 months	1975	86.2	0–11 months	494	72.1
					≥12 months	78	11.4
		≥12 months	317	13.8	0–11 months	43	6.3
					≥12 months	70	10.2
		N =	2292		N =	685	
	≥12 months	0–11 months	329	38.6	0–11 months	60	24.3
					≥12 months	30	12.1
		≥12 months	524	61.4	0–11 months	38	15.4
					≥12 months	119	48.2
		N =	853		N =	247	

a Children being breastfed at the time of the survey who had not reached the breastfeeding duration target were classed as ‘unknown’ and were excluded from these analyses. When breastfeeding had ceased women and children were included regardless of the child’s age.

## Discussion

Consistent with previous findings [2], [6], we have shown low rates of breastfeeding in Australian infants to the six, 12 and 24 month duration targets. Only 60% of nearly 10,000 infants received the minimum recommended 6 months of breast milk, irrespective of breastfeeding exclusivity. Less than 30% of infants received any breast milk at 12 months, and less than 3% were breastfed to the international target of 24 months.

While national breastfeeding rates are typically evaluated in relation to the infant, a novel component of our study is that we have also assessed maternal adherence to breastfeeding duration targets and the longitudinal feeding practices of women with more than one child. About half of the mothers in the 1973–78 ALSWH cohort breastfed all of their children for six months or more. Only 20% of mothers breastfed all of their children for 12 months or more, and less than 2% of mothers breastfed all their children to 24 months or more. For women with three or more children, nearly 75% of women who breastfed their first child for at least six months reached this breastfeeding duration target for their next two children. On the other hand, half of the mothers who did not reach the six month duration target with their first child also did not breastfeed their subsequent children to six months or more. Our data highlight the importance of helping women to establish good breastfeeding practices with their first child, so that these can be continued for any subsequent births. The findings also emphasise the importance of developing appropriate interventions to increase breastfeeding of second and subsequent children for women who did not meet breastfeeding duration targets with their first child.

Separate evaluations of maternal and infant breastfeeding rates are important as they have different implications for public health policy and practice. The number of infants being breastfed is important particularly in relation to the known health outcomes, such as the reduction in infectious diseases [2]. The economic benefits of breastfeeding relate to health-care costs, productivity and household expenses [2]. The rates of infant breastfeeding also provide measures to compare against national targets and data from other countries. However, the profiles of women who adhere or do not adhere to the breastfeeding duration targets are equally important from a resource allocation and intervention perspective. Ultimately interventions aimed at improving national breastfeeding rates must be either targeted at, or mediated through, the mother.

Our study, like others [13], has shown that older mothers, who are married, well-educated and/or are not stressed about money are more likely to breastfeed for longer durations. We have also shown that infants who are full term and not low birth weight, with older siblings, are most likely to be breastfed at least to 12 months or more. Health professionals may need to go to greater lengths to support young, less educated, unmarried or low-income women, and those with preterm or low birth weight infants, who are at an increased risk of premature breastfeeding cessation. Appropriate breastfeeding interventions should be tailored and tested specifically for women who need it most. This includes targeting first-time mothers, helping them to establish a good breastfeeding track-record. Self-efficacy has been described as an important predictor of breastfeeding duration. In an Australian study, Blyth et al. (2002) showed primiparous women had significantly lower self-efficacy scores compared to multiparous women with previous breastfeeding experience. Interestingly, self-efficacy was not associated with maternal age, marital status, education or ethnicity [14], and hence may offer an intervention target for all women.

The data in this study were collected prior to the Australian Government’s introduction of the first Paid Parental Leave Scheme; 18 weeks paid at the national minimum wage, which commenced on 1 January 2011. This federal scheme forms part of the *Australian National Breastfeeding Strategy 2010–2015* aimed at encouraging women, particularly those who are employed, to breastfeed exclusively for longer [15]. Bonet *et al.* (2012) recently showed that women who returned to work within four months of a birth were less likely to continue breastfeeding than women who went back to work later, regardless of full- or part-time employment status [16]. Our data can serve as a pretest reference for Australia’s paid parental leave scheme.

The strengths of our study include the large number of children born to the broadly nationally representative sample of Australian women, who were aged 31–36 years at the time of the survey. This cohort has reported their breastfeeding data for any prior live birth. However, data are also limited because the maternal characteristics do not match with when the child was actually breastfeeding but when the data were collected. Furthermore, they do not capture breastfeeding by women older than 36 years. Generally, women who had their first child later were more likely to adhere to the breastfeeding duration targets and being later in the birth order was also associated with being breastfed for longer. With 15% of women in Australia having their first birth after the age of 35 years [17], we may have underestimated the true proportion of infants breastfed in line with current duration targets. However, our rates of any breastfeeding to 6 months are almost identical to the most recent figures published in the 2010 *National Infant Feeding Survey* [6]. Breastfeeding rates in Australia reached their nadir in the early 1970s, but had recovered by the 1980s [15] when the 1973–78 cohort was first capable of reproduction. Since then, data [18] and experts suggest that breastfeeding rates have remained fairly static [15], reducing the likelihood of time as a confounder.

Another limitation of our self-reported data is the potential for recall bias, which is known to increase as time from the event increases. Li *et al.* (2005) conducted a systematic review of 11 studies on the validity and reliability of maternal recall of their breastfeeding practices. For mothers with a recall period of three years or less, the majority accurately recalled their duration of breastfeeding to within 1 month [19]. Within the ALSWH cohort the average time since the last breastfeeding episode was 2.8 years. One other study showed that many years after breastfeeding had ceased, women who recorded short durations of breastfeeding tend to over-report their breastfeeding durations, while women with long durations tend to under-report [20].

### Conclusion

Australian infants are not being fed in accordance with the national and international breastfeeding duration targets. While reporting the rates of infant breastfeeding is important, so too is an assessment of mothers’ adherence to breastfeeding practices. This is especially true when you consider that it is the mother responsible for delivering the feeding practice, and this practice is likely to be provided to more than one child over her childbearing years. While the socio-demographic maternal and child characteristics associated with breastfeeding duration are not modifiable at the point of service, it is important for health professionals and policy makers to be aware of the groups at high-risk of inadequate breastfeeding. In this way appropriate interventions can be designed and tested, with the goal of improving breastfeeding and thereby the health of the population.
